# Identifying artemisinin resistance from parasite clearance half-life data with a simple Shiny web application

**DOI:** 10.1371/journal.pone.0177840

**Published:** 2017-05-22

**Authors:** Sai Thein Than Tun, Yoel Lubell, Arjen M. Dondorp, Tom Fieldman, Kyaw Myo Tun, Olivier Celhay, Xin Hui Chan, Sompob Saralamba, Lisa J. White

**Affiliations:** 1 Mahidol-Oxford Tropical Medicine Research Unit, Mahidol University, Bangkok, Thailand; 2 Nuffield Department of Medicine, University of Oxford, Oxford, United Kingdom; 3 School of Clinical Medicine, University of Cambridge, Cambridge, United Kingdom; 4 Defence Services Medical Academy, Yangon, Myanmar; Quensland University of Technology, AUSTRALIA

## Abstract

The emergence of artemisinin-resistant *Plasmodium falciparum* malaria is a major threat to malaria elimination. New tools for supporting the surveillance of artemisinin resistance are critical for current and future malaria control and elimination strategies. We have developed an open-access, user-friendly, web-based tool to analyse parasite clearance half-life data of *P*. *falciparum* infected patients after treatment with artemisinin derivatives, so that resistance to artemisinin can be identified. The tool can be accessed at bit.ly/id_artemisinin_resistance.

## Introduction

The prospect of malaria elimination is under threat due to the emergence of parasites resistant to artemisinin derivatives, the most efficacious drugs against *Plasmodium falciparum* malaria [1]. The therapeutic efficacy of artemisinin-based combination therapy (ACT) has to be monitored every two years in order to respond to resistance [2]. With improving methods for genotyping, such as the K13 molecular marker, the working definition of artemisinin resistance has been gradually adapted over the past few years [2]. In the latest WHO recommendations [2], the cut-off value of ≥ 10% of patients with a half-life of parasite clearance slope ≥ 5 hours after treatment with ACT or artesunate monotherapy, is used as one of the definitions of “suspected endemic artemisinin resistance”. White et al [3] developed a model to analyse data on parasite half-life as distributions of artemisinin-sensitive and artemisinin-resistant populations, which could help in the surveillance of artemisinin resistance. The model has been validated using parasite half-life data from studies on the Thai-Myanmar border and in Western Cambodia.

The source code of the model, developed in the R programming language[4] by White et al., is available on Github [5]. Interested parties have been using the model to analyse their own parasite half-life data. Until now, this has been limited to modellers proficient in the R programming language[4]. We have developed a user-friendly interface which will make this model available to a wider audience.

## Methods and implementation

For a given set of half-life measurements, mixture models can be used to detect and describe subgroups of individuals or subpopulations, if there are any, without having any information on the membership of each individual observation [6]. White et al. [3] used this mixture modelling approach to estimate the geometric mean, standard deviation, and the relative contributions of each subpopulation, given the observed population’s half-life data. Mixture models with a range of one to five components were fitted to the data, and the most suitable model was chosen. By applying this method to parasite clearance half-life data from the Thai-Myanmar border and Western Cambodia, two subpopulations, which represent artemisinin sensitive and artemisinin resistant populations, were detected. This method was also validated by comparison with the parasite half-life distributions of clonal parasite infections. For details of this method, please refer to the original paper [3].

**Fig 1:**
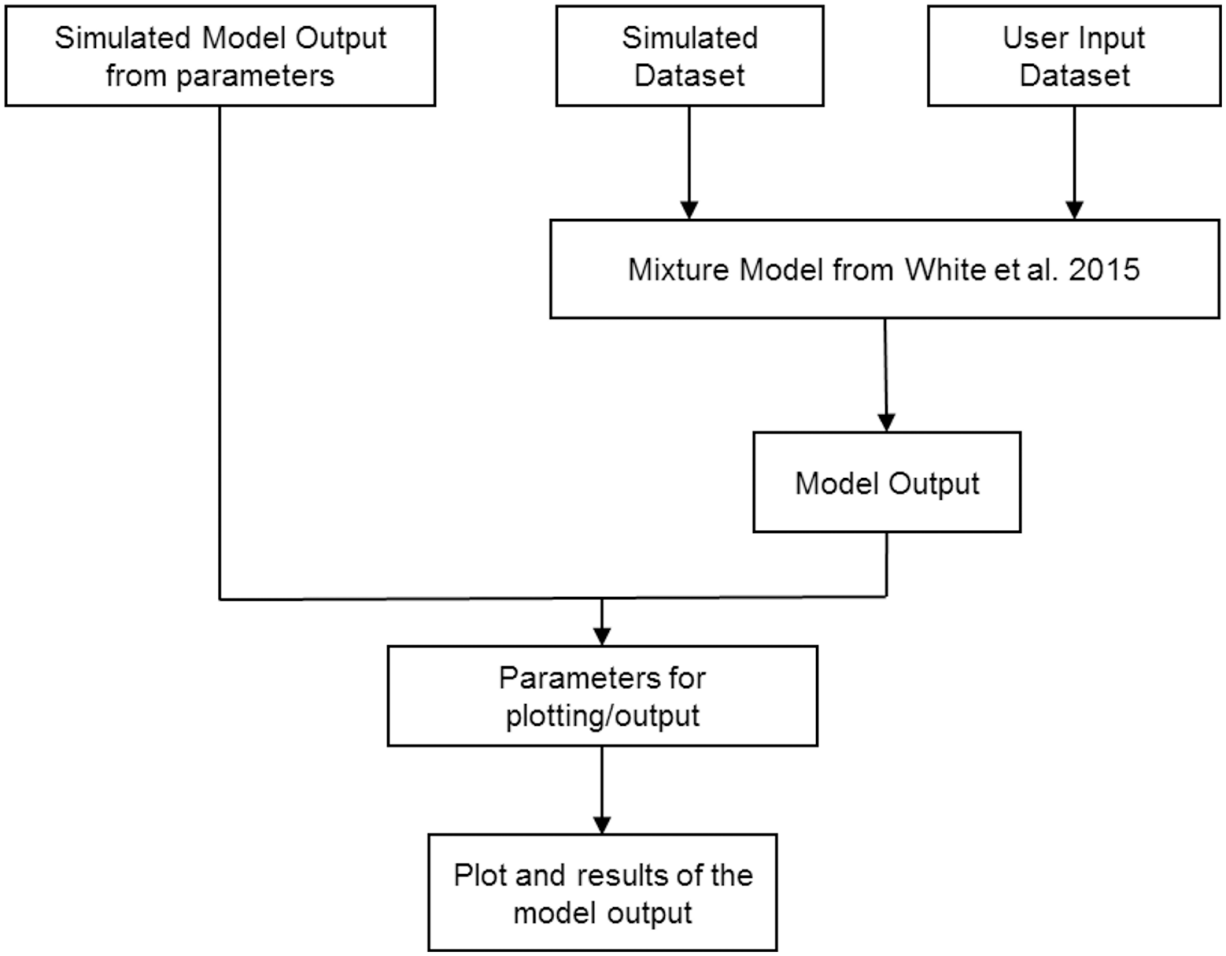
Workflow of the Shiny web application (bit.ly/id_artemisinin_resistance).

The source code of the model, written in the R programming language [4], was provided in the original paper [5]. We extracted the specific code for the mixture models with log-normal distribution. Shiny [7], an R package used to build interactive web applications directly from R scripts, was utilized to modify the code to produce an interactive user interface for the mixture model from White et al. The other R packages used were mixtools [6], pROC [8], MASS [9], shinythemes [10], and shinydashboard [11]. The workflow of the web application is described in Fig 1.

**Fig 2:**
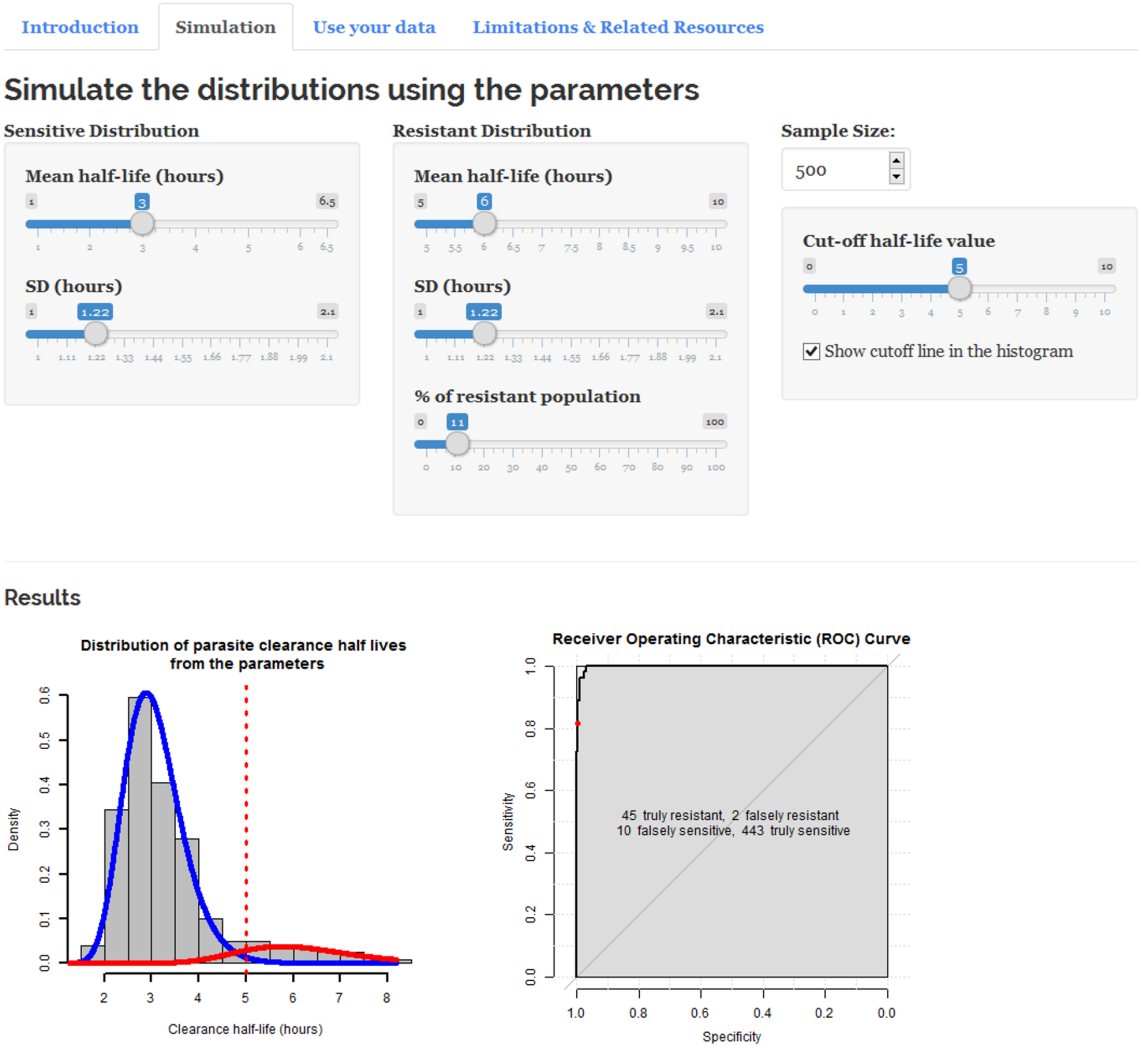
A section of the user interface of the Shiny web application. (bit.ly/id_artemisinin_resistance).

There are three main sections on the user interface of the web application. The Introduction describes why this tool should be used. It also provides two hypothetical examples which users can toggle by clicking on the respective buttons. In the “Simulation” section, users may change the parameters to generate the multimodal composite distributions themselves. This will help users to understand more about the model and the multimodal distributions. Part of the user interface can be seen in Fig 2.

**Fig 3:**
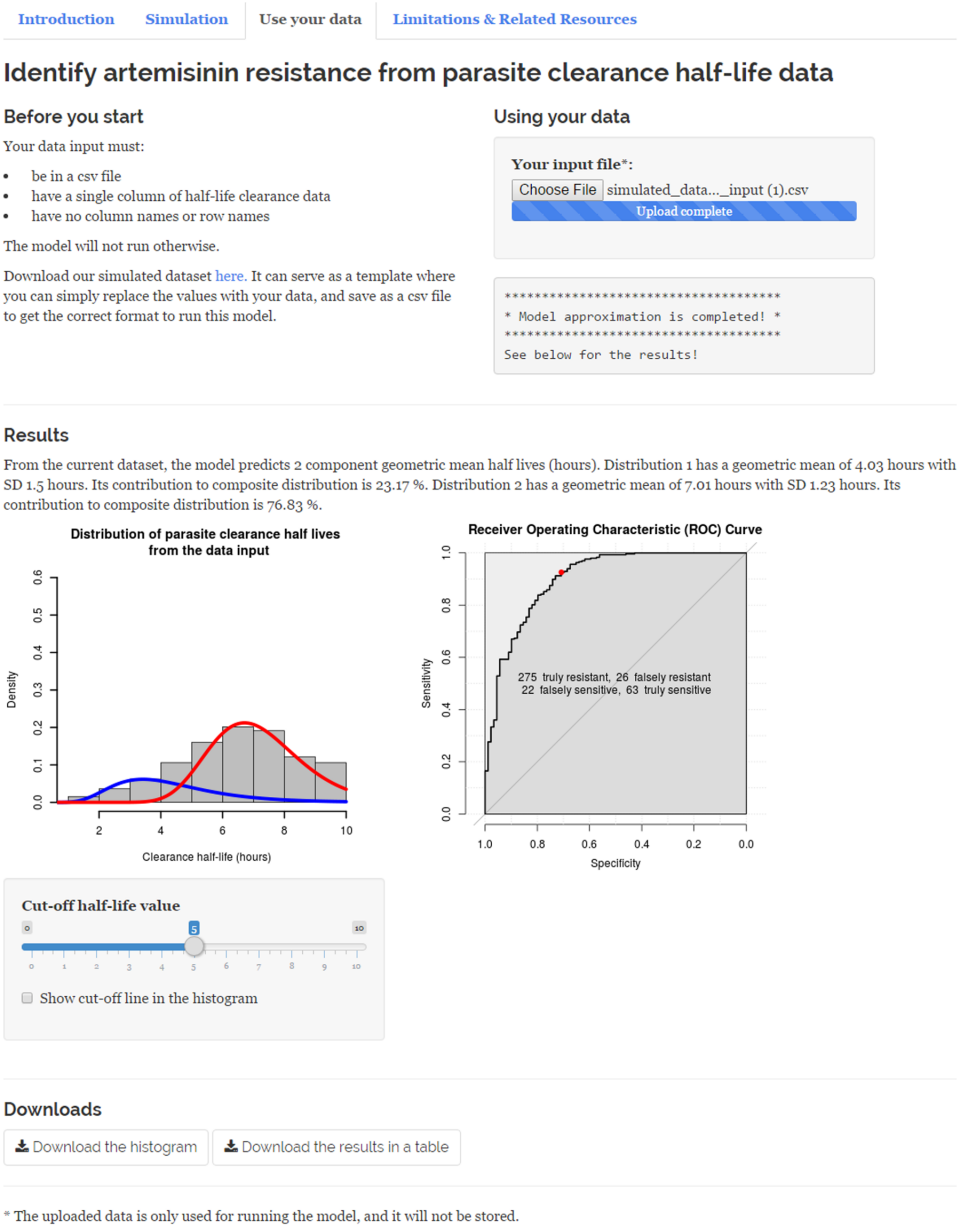
“Use your data” section of the Shiny web application (bit.ly/id_artemisinin_resistance) with a sample result.

In the "Use your data" section, a simulated dataset can be downloaded as an example of the format of the data which is required for running the model. This dataset can also serve as a template onto which users can overwrite (copy and paste) their own half-life data. The input dataset must be in comma-separated values (CSV) file format, with a single column of half-life clearance data and without column and row names. Once the users have their half-life data in the correct format, it can be uploaded with the "Upload File" button. The model will process the data in the background, which will take from a few seconds to a few minutes depending on the size of the data input. For example, the simulated dataset we have provided has 388 rows of data and it takes approximately 20 seconds to process. Users will be notified once the process is completed or if the input dataset is not in a correct format. Fig 3 shows the “Use your data” section with the result of the model from processing the simulated dataset. There are two download buttons at the bottom of the page: one to download the graph and another one to download the results as a table in CSV file format. If the model estimates two subpopulations, where the one with shorter half-lives represents the sensitive population and the other with longer half-lives represents the resistant population, then a Receiver Operating Characteristic (ROC) curve will be plotted. From this, it can be discerned how well a particular cut-off value of half-life performs in detecting resistance, given the assumption that each unimodal distribution represents infections with a subpopulation of parasites.

We host the web application on the Shiny server at this web address (bit.ly/id_artemisinin_resistance) and the corresponding source code at (bit.ly/White-2015-shiny-code). The web application was tested and found to be working on the following internet browsers: Mozilla Firefox (v48.0.2), Google Chrome (v53.0), Internet Explorer (v11.0), Safari (v9.0.1), Opera (v39.0), Maxthon (v4.9.3), and the mobile version of Google Chrome (v52.0). The only requirement to use this web application is access to the internet.

## Results and discussion

We developed an open-access interactive web application based on the model by White et al [3], which can be found at this address: bit.ly/id_artemisinin_resistance. It can analyse parasite clearance half-life data as distributions of drug-sensitive and drug-resistant populations. This is a potentially useful tool for the surveillance of artemisinin resistance, and could be used alongside other methods such as the identification of molecular markers. It can also be used to measure the probability of a parasite strain being artemisinin resistant for an infection with a particular parasite clearance half-life.

We also demonstrated how a complicated model could be adapted into a user-friendly web application. Since an internet connection is the only requirement, there is significant potential for such modelling tools to be used by and presented to a wider audience.

### Limitations

All of the assumptions from the original paper [3] are applied here. The clearance half-lives of infections with a particular sensitivity are assumed to follow unimodal distributions of log-normal type. The model’s accuracy will be reduced with an increasing number of true components in the distribution and decreasing sample size. For instance, with a sample size of 1,000, the model will correctly predict 96%, 91%, 70%, 46% and 21% for the input mixture distributions of 1, 2, 3, 4 and 5 components respectively. From a sample size of 50, the model will be able to differentiate between subpopulations of geometric mean half-lives with a difference of 3 or more hours. From a sample size of 1000, the model will be able to differentiate subpopulations whose geometric mean half-lives differ by only 0.5 hours.

The web application will not work if the input data is not in an appropriate format. In that case, it will display an error message. At the time of preparation of this manuscript, the web application has been hosted on a Starter version of Shiny server, which allows 100 hours of active usage per month with premium support. Since the Shiny server is easily scalable, further upgrades can be considered in case of heavier traffic to the web application. We have committed to maintain this web application over the next 5 years, after which time its access may be limited to 25 active hours per month, which is standard for a free Shiny account.
